# The relationship between visual memory and spatial intelligence with students’ academic achievement in anatomy

**DOI:** 10.1186/s12909-023-04327-9

**Published:** 2023-05-16

**Authors:** Amin Aspanani, Hosein Sadeqhi, Athar Omid

**Affiliations:** 1 School of Medicine, Isfahan University of Medical Sciences, Isfahan, Iran; 2 Department of Anatomical Sciences, School of Medicine, Isfahan university of medical sciences, Isfahan, Iran; 3 Medical Education Research Center, Department of Medical Education, Isfahan University of Medical Sciences, Isfahan, Iran

**Keywords:** Visual memory, Spatial intelligence, Medical students, Dental students

## Abstract

**Introduction:**

Academic achievement is influenced by various factors. Spatial intelligence and visual memory are among the factors that seem to be related to learning anatomy. The aim of this study was to investigate the relationship between visual memory and spatial intelligence with students’ academic achievement in anatomy.

**Methods:**

The present study is a descriptive cross-sectional study. All medical and dental students who had chosen anatomy courses (Semester 3 medicine and 2 dentistry) were the target population (*n*=240). The study tools were Jean-Louis Sellier 's visual memory test to determine visual memory and ten questions from Gardner Spatial Intelligence Questionnaire were employed to assess spatial intelligence. The tests were performed at the beginning of the semester and its relationship with the academic achievement scores of the anatomy course was examined. Data were analyzed by descriptive statistics, independent t-test, Pearson correlation and multiple linear regression.

**Results:**

Data of 148 medical students and 85 dental students were analyzed. The mean score of visual memory in medical students (17.1±5.3) was significantly higher than dental students (14.3±4.6) (*P*-value <0.001). But the mean score of spatial intelligence (31.5±5.9) was not significantly different between medical and dental students (31.9±4.9) (*P*-value=0.56). Pearson correlation coefficient showed that in medical students there was a direct relationship between visual memory score and spatial intelligence score with scores of anatomy courses (*P*-value<0.05). Moreover, in dental students, there was a direct relationship between the score of anatomical sciences with the score of visual memory (*P*-value=0.01) and the score of spatial intelligence (*P*-value=0.003).

**Conclusion:**

The results of this study showed that there is a significant relationship between spatial intelligence and visual memory with learning anatomy and planning to enhance these characteristics can be fruitful in students. It is suggested that Visual memory and spatial intelligence should be considered for student admission, especially in the fields of medicine and dentistry.

## Background

Academic accomplishment is one of the most important concerns of any educational system. Success and academic achievement show the efficiency of the educational system in the field of goal setting and attention to meeting individual needs. Therefore, the study of factors affecting academic achievement has received more attention in recent years [1]. These influential factors include cognitive skills such as spatial intelligence.

Spatial intelligence is the ability to analyze an object in three-dimensional space and draw conclusions from limited information [2]. Spatial ability, as the most important cognitive ability, enables us to visualize an object or a space, manipulate a scene mentally , animate, rotate, and resize an object in space [3, 4]. This ability is considered necessary and important in all STEM (Science, Technology, Engineering and Mathematics) disciplines. As we know in STEM (Science, Technology, Engineering, & Mathematics) disciplines, current medical education curriculum aims to enhance the students' multiple intelligences and students can make transition from being a novice to a master [5, 6]. In the Cattell-Horn-Carroll model of intelligence, this classifies under eleven factors: visualisation, speeded rotation, closure speed, flexibility of closure, visual memory, spatial scanning, serial perceptual integration, length estimation, perceptual illusions, perceptual alternations, and Imagery [7]. Visual memory is among several cognitive systems which preserves some characteristics of our senses pertaining to visual experience [8]. Visual memory is responsible for storing pictures and printed text impinging on the eyes as exact visual images and is part of the sensory memory system [9]. However, academically memory and spatial intelligence are invariably taken as the criteria for students’ academic achievement and progress in medicine and engineering [10, 11].

One of the main courses in medical sciences is anatomy. Prerequisite for academic achievement in anatomy is to learn many spatial concepts, such as the shape of anatomical structures, their relative locations, and how they are connected. Most of the research related to academic achievement in this course is related to the usage of different educational methods such as virtual education and flipped classroom [12, 13] and the effects of technology such as extended reality and 3D printing on education [14, 15]. But research on spatial abilities and their factors, such as visual memory, has been limited.

In a study by Aydin et al, it was found that there is significant relationship between students’ spatial ability and their success in practical anatomy examinations [16] and Vorstenbosch et al. found that learning anatomy could enhance spatial ability [17]. A 2011 study by Fernandez et al. compared spatial ability in novices, intermediates, and experts in anatomy. They found that experts and intermediates had better spatial ability than novices and education and experience contribute to further development of these abilities [18].

In general, research on the relationship between spatial intelligence and visual memory in anatomy education has been limited. The existing studies have mostly examined the relationship between spatial intelligence and anatomy learning. No study has been found to investigate the relationship between both spatial intelligence and visual memory simultaneously with medical and dental student's academic achievement in anatomy. Therefore, this study was conducted to investigate the relationship between visual memory and spatial intelligence with anatomy learning at the Isfahan University of Medical Sciences.

## Methods

The present study is a cross-sectional study (descriptive-correlation). In this study, the aim was to determine the correlation between visual memory and spatial intelligence with academic achievement in anatomy courses at the Isfahan University of Medical Sciences. The study population was 3^rd^ year medical students who were taking practical head and neck anatomy, theoretical head and neck anatomy, neuroanatomy and special senses and 2^nd^ semester dental students who were taking an anatomy science course. The study was conducted with the population of 240 people.

All 3^rd^ year medical students who were taking practical head and neck anatomy, theoretical head and neck anatomy, neuroanatomy and special senses and second-semester dental students who were taking an anatomy course and wanted to participate in the study were included in this study. Exclusion criteria included: failure to complete the questionnaire or its distortion was the tendency to leave the study, failure to participate in the relevant course exams and the effect of disciplinary cases on the final course grades.

**Fig. 1:**
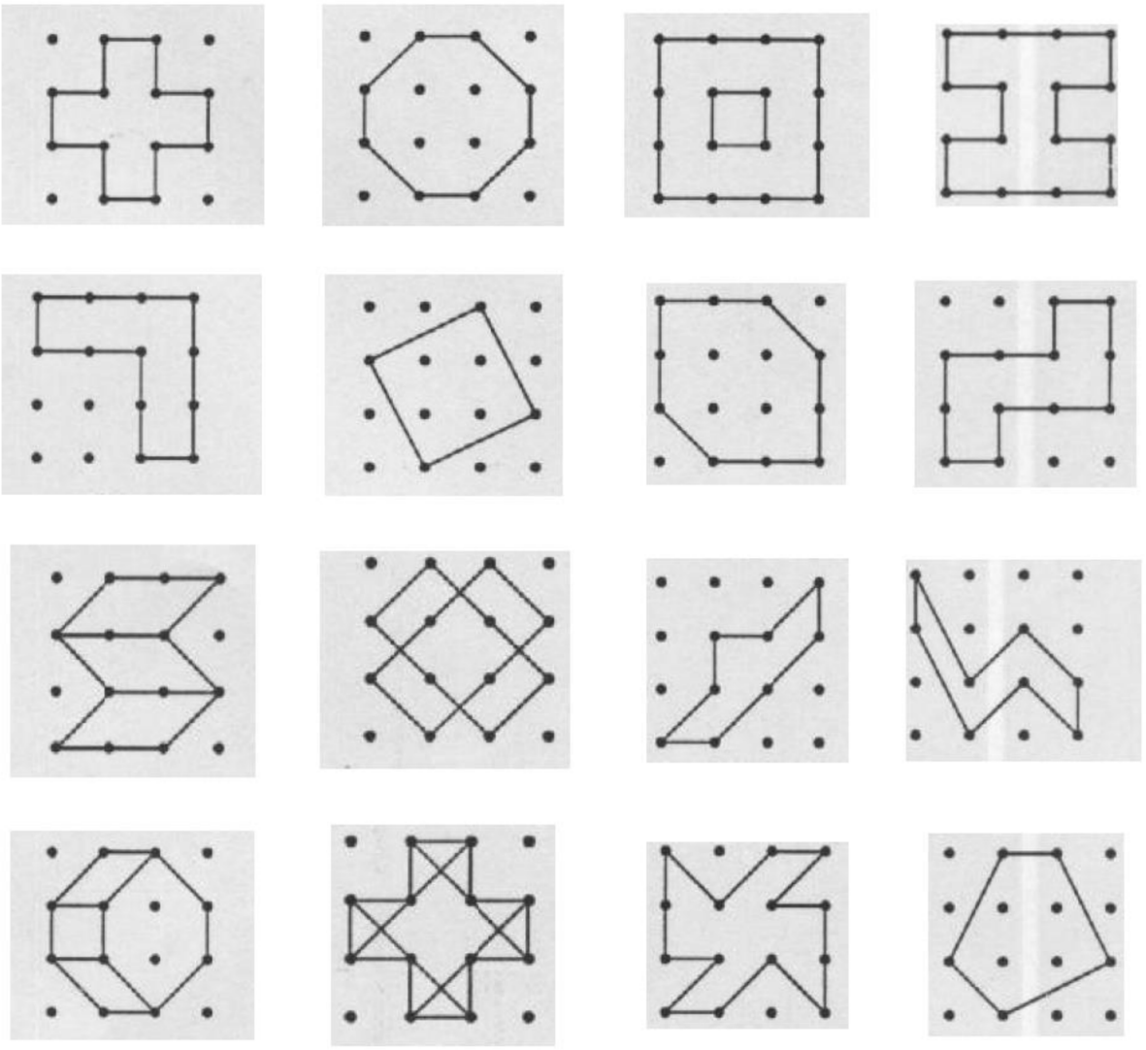
Jean-Louis Sellier visual memory test: The participants have to look at all the pictures and memorize them, and then draw them without looking at the pictures [19]

In this study, two questionnaires of Jean-Louis Sellier (to assess visual memory) and Gardner questionnaire (to assess spatial intelligence) were used. The Jean-Louis Sellier Questionnaire was developed in the 1970s by Jean-Louis Sellier in France [19]. This tool is a performance test with which fluid factors, expansion, flexibility and originality can be measured and a total score for visual memory can be calculated. This tool was first introduced by Dr. Hadi Bahrami Ehsan in Iran and Khodabakhshi Koolaee declared the reliability of this test through Cronbach's alpha of 0.81 [20]. The material of this test includes 16 images in 16 positions (Fig. 1). Images are made up of smooth lines that run through points on the paper. First, the participants drew some pictures to practice by looking at the main pictures. In the main stage of the test, the participants looked at all 16 pictures and memorized them, and then they had to draw without looking at the pictures. The scoring method is such that the correct drawing of each shape in the correct position has 2 points. If the shape is correct but the position of the shape is wrong, 1 point and if the shape is wrong, no points will be awarded. The final score of this test can be numbers between 0 and 32 which is the sum of the points obtained by the examiner. It took about10 minutes to memorize pictures and 10 minutes to draw pictures in the main stage of the test.

The Multiple Intelligences Questionnaire was developed by Gardner in 1983 [21]. This 80-item questionnaire measures eight types of intelligence. The answers to this questionnaire are scored in a 5-point Likert scale from 1 = very low to 5 = very high. TIRRI presented the latest version of the Multiple Intelligences Profiling Questionnaire (MIPQ III) that is based on Howard Gardner’s (e.g., 1983, 1999) MI theory. Five-point Likert scale is also used in this questionnaire and it is a selfrating questionnaire [22]. In this study, the Persian version of the questionnaire that was presented by Azarfar was used [23]. According to the needs of this study, only 10 designed questions were to assess spatial intelligence. Azarfar and Niroo et al reported the reliability each of the eight multiple intelligences categories based on Cronbach's alpha index (Alpha Chronbach spatial intelligence questionaire= 0.81) [23, 24]. Malekian and Maleki also obtained the reliability of Gardner questionnaire 0.80 [25]. Student's academic achievement at the end of the school year was assessed based on the final grades in each course. These scores included numbers for each lesson with scores ranging from 0 to 20. This information was received from the training centers of each faculty.

The questionnaire was distributed among the students of the third semester of medicine and the second semester of dentistry by the researcher at the beginning of the academic semester (October 2017). Students completed the questionnaires on their own and they were guided by the researcher. At the time of completing the questionnaires, students were explained that the test results would not affect their grades in any way. The whole process of extracting information was clarified to individuals; They were told that the only personal information needed from the people was their student number and gender and If you wish to participate in the research, write your student number and gender on the test sheets. If they are not satisfied with the required information, they can leave it at any stage of the research. It was also mentioned that the results would be analyzed and published in general without providing personal information and even their student number. While being completed the questionnaire, the time and environmental conditions were the same for everyone. Out of 240 people in the target population, 235 people agreed to participate in the study. After collecting the tests due to the distortion of the questionnaire, 2 people were excluded from the study. Finally, information was extracted from 148 medical students and 85 dental students. At the end of the academic year, after submitting a commitment to the university educational centers to keep information confidential. The final grades of the students participating in the research were received only with their student number.

With use of student's numbers, lesson scores were matched with the test results for analysis. All statistical studies were performed in two sections: descriptive and analytical. In the descriptive part, the reports were presented as mean (standard deviation) for quantitative variables and frequency percentage (number) for qualitative variables. Univariate correlation between variables was performed using Pearson correlation coefficient based on statistical assumptions. Data were entered in SPSS20 and analyzed by descriptive statistics, independent t-test, Pearson correlation and multiple linear regression.

## Results

Among medical students, 52.7% were male, and among dental students 44.7% were male. The mean scores of visual memory and spatial intelligence in male and female dental students are given in Table 1. Independent t-test showed that the mean scores of visual memory (*P*-value=0.14) and spatial intelligence (*P*-value =0.66) in dental students were not significantly different between men and women.

**Table 1:** Mean scores of visual memory and spatial intelligence in male and female students in dentistry

Field	Variable	Male	Female	*P*-value
		Mean±SD	Mean±SD	
Dentistry	Visual memory	15.1±4.3 *N*=38	13.6±4.9 *N*=47	0.14
	Spatial intelligence	32.2±4.4 *N*=38	31.8±5.2 *N*=47	0.66
Medical	Visual memory	17.8±5.1 *N*=78	16.3±5.3 *N*=70	0.08
	Spatial intelligence	31.4±6.5 *N*=78	31.7± 5.2 *N*=70	0.76

A significance level of less than 0.05 is considered

The mean scores of visual memory and spatial intelligence in male and female medical students are given in Table 1. Independent t-test showed that the mean scores of visual memory (*P*-value=0.08) and spatial intelligence (*P*-value=0.78) in medical students did not differ significantly between men and women.

The mean scores of visual memory and spatial intelligence in students of the two groups are given in Table 2. The mean and standard deviation of visual memory in medical students was 17.1±5.3 and in dental students was 14.3±4.6. Independent t-test showed that the mean score of visual memory in medical students was significantly higher than dental students (*P*-value<0/001). The mean score and standard deviation of spatial intelligence in medical students was 31.5±5.9 and in dental students was 31.9±4.9. Independent t-test demonstrated that the mean score of spatial intelligence was not significantly different between students of the two disciplines (*P*-value=0.56).

**Table 2:** Mean and standard deviation of visual memory and spatial intelligence scores in students of two disciplines

	Medical	Dentistry	
Score	Mean and Standard deviation	Mean and Standard deviation	*P*-value
Visual memory	17.1±5.3	14.3±4,6	>0.001
	*N*=148	*N*=85	
Spatial intelligence	31.5±5.9	31.9±4,9	0.56
	*N*=148	*N*=85	

A significance level of less than 0.05 is considered

The relationship between visual memory scores and spatial intelligence in each of the two groups showed that there was a weak positive relationship between spatial intelligence score and visual memory score in students of both disciplines (*P*-value<0/001 & ${r}_{dentist}=0.280 {r}_{medical}=0.294$). In other words, as the visual memory score increased, so did the spatial intelligence score (Table 3).

**Table 3:** Pearson correlation coefficients between visual memory and spatial intelligence scores in each of the two groups

Variable	field	Visual memory	
		R	*P*-value
Spatial intelligence	Medical	0.294	>o.oo1
	*N*=148		
	Dentistry	0.280	>o.oo1
	*N*=85		

A significance level of less than 0.05 is considered

The relationship between visual memory scores and spatial intelligence with the scores of different courses as well as the average final scores of each course in medical students is shown in Table 4. Pearson correlation coefficient showed that in medical students there was a direct relationship between visual memory score and spatial intelligence score with scores of neuroanatomy courses, special senses, theoretical head and neck and practical head and neck (*P*-value<0.005). The relationship between visual memory and spatial intelligence scores with the score of anatomical sciences course as well as the average final scores in anatomical sciences in dental students is given in Table 4. Pearson correlation coefficient showed that in dental students there was a direct relationship between the score of anatomical science theory and the score of visual memory (*P*-value=0.01 & *r*=0.262) as well as the score of spatial intelligence (*P*-value =0.003 & *r*=0.319) .

**Table 4:** Pearson correlation coefficients between visual memory and spatial intelligence scores with scores of different courses in medical and dental students

Field		Average grade	Visual memory		Spatial intelligence	
			r	*P*-value	r	*P*-value
Medical	Neuroanatomy	15.74	0.282	0.001	0.209	0.01
	*N*=148					
	Special Senses	14.39	0.238	0.004	0.233	0.004
	*N*=148					
	Head and neck theory	14.62	0.239	0.003	0.229	0.005
	*N*=148					
	Practical head and neck	17.52	0.293	>0.001	0.291	>0.001
	*N*=148					
Dentistry	Anatomical science score	15.45	0.262	0.01	0.319	0.003
	*N*=85					

A significance level of less than 0.05 is considered

Linear regression analysis to predict the score of anatomy course based on the scores of visual memory and spatial intelligence by field of study is shown in Table 5. Multiple linear regression analysis showed that in medical students as well as dental students, visual memory and spatial intelligence scores were significant predictors of anatomy lesson scores. In medical students, visual memory scores were better predictors of anatomy scores, but in dental students, spatial intelligence scores were better predictors of anatomy scores. In this study, the reliability of Jean-Louis Sellier's test was 0.69 through Cronbach's alpha and the reliability of Gardner space intelligence questionnaire was 0.62 through Cronbach's alpha.

**Table 5:** Linear regression analysis to predict anatomy course score based on visual memory and spatial intelligence scores by field of study

Field	Score	Raw coefficients	Standardized coefficients	t	*P*-value
Medical	Visual memory	0.108	0.246	3.04	0.003
	*N*=148				
	Spatial intelligence	0.083	0.211	2.62	0.01
	*N*=148				
Dentistry	Visual memory	0.092	0.187	2.05	0.04
	*N*=85				
	Spatial intelligence	0.125	0.267	2.49	0.02
	*N*=85				

A significance level of less than 0.05 is considered

## Discussion

Based on the results of the study, a significant relationship was observed between visual memory and spatial intelligence and academic scores in both medical and dental groups and people with higher visual memory and spatial intelligence had higher academic scores. Multiple regression analysis also showed that visual memory and spatial intelligence are significant predictors of student's academic achievement in anatomy courses. “Sweeney et al.” found in a study that there is a weak correlation between the outcome of spatial assessments and student's performance in learning anatomy, but spatial learning ability is a predictor of initial success in anatomy learning [26]. Lufler et al.'s study also found that students with higher spatial-visual abilities were more successful in both practical tests and end-of-course theory [27]. Langlois et al in a systematic review and Roach et al also in a meta‐analysis found that there are no significant relationships between spatial abilities test and anatomy knowledge assessment using essays and non-spatial multiple-choice questions but there are significant relationships between spatial abilities test and anatomy knowledge assessment using practical examination and drawing tasks [28, 29]. The present study showed that medical students had better visual memory than dental students. While there was no significant difference in the level of spatial intelligence between students of the two groups. In a study by Vorstenbosch et al., two groups of 242 medical students and a control group of 258 social science students were selected. The results of pre-test evaluation showed that medical students have a significantly higher spatial ability than social science students [30].

Other results of the present study showed that the level of spatial intelligence and visual memory in both groups of medical and dental students did not differ significantly between the sex groups of girls and boys. This is something seen in the study of "Aymeric Guillot et al" which examined the relationship visuo-spatial representation and mental rotation with learning anatomy. They realized that visuo-spatial representation and mental rotation abilities of male students were higher than female ones, but there was a weak relationship between these cases in both sexes with learning anatomy [31]. The results of this study are contrary to Roach et al study which showed that there was a significant sex difference in spatial ability, and males had better perfromance than females on spatial ability tasks. This difference is perhaps due to the difference in the type of study, which is more valid in meta-analysis [29]. One of the strengths of the present study is the use of high sample size. In this study, paying attention to the morale of the participants and the effect of environmental conditions on students when completing the questionnaires, especially the Jean-Louis Sellier test, was very important and could affect the results. To solve this problem, an attempt was made by unifying the conditions for all students when completing the exams and assuring them that their course grades were not surely going to be affected by the test and also the principle of secrecy.

## Conclusion

According to the results of this study, there is a relationship between the level of spatial intelligence and visual memory with student's academic achievement in anatomy courses. In addition, this study showed that there is a significant positive correlation between visual memory and spatial intelligence in both groups of medical and dental students. Thus, students with high visual memory also have higher spatial intelligence. Therefore, it is suggested to plan to strengthen these features in learners. Efforts to enhance spatial intelligence and visual memory can be made in universities, but it may be more effective to start such interventions at an earlier age. “Ghadamzan & Nazeri” in a study showed that teaching painting to preschool children has an influence on increasing children's visual-spatial intelligence and quantitative findings showed this effect to be up to 21% [32]. Educational centers, especially elementary schools, are suggested to increase such interventions to strengthen student's spatial intelligence and visual memory. Based on the results of this study and of the others’ studies such as Wai et al and Lubinski, it can be recommended visual memory and spatial intelligence could be incorporated in the academic guidance of students, whose disciplines are experimental, like student admission and talent identification, especially in medicine and dentistry [33, 34].

## Data Availability

The datasets used and/or analyzed during the current study available from the corresponding author on reasonable request and all relevant data are included in the article.
